# CT and MRI findings in relapsing primary malignant melanoma of the lacrimal sac: a case report and brief literature review

**DOI:** 10.1186/s12886-020-01356-6

**Published:** 2020-05-14

**Authors:** Ju-Wei Shao, Jian-Hua Yin, Shu-Tian Xiang, Qian He, Hong Zhou, Wei Su

**Affiliations:** 1 Department of Radiology, the Fourth Affiliated Hospital of Kunming Medical University, 176 Qing Nian Street, Kunming, People’s Republic of China; 2 Department of Radiology, the First Hospital of Kunming, Kunming, People’s Republic of China; 3 Department of Pathology, the Fourth Affiliated Hospital of Kunming Medical University, Kunming, People’s Republic of China

## Abstract

**Background:**

Primary lacrimal sac melanoma is an extremely rare condition with fewer than 50 cases reported so far. Clinically, its symptoms resemble those of dacryocystitis, leading to frequent misdiagnosis. During diagnosis, imaging examination is often performed to differentiate tumors from inflammation. In this report, we present a case of primary lacrimal sac melanoma and summarize the CT and MRI characteristics of lacrimal sac melanoma.

**Case presentation:**

We report a 50-year-old female patient who had undergone a dacryocystectomy for the left lacrimal sac mass. Postoperative pathological examination confirmed the presence of primary malignant melanoma. Three months later, a lump in the lacrimal sac area was found. The patient underwent CT and MR examinations. CT scan demonstrated a partially well-defined soft mass in the fossa of left lacrimal sac extending into the nasolacrimal duct and anterior ethmoid sinus. MRI revealed an intermediate signal intensity on T1 and iso-or hyper-signal on T2 weighted images. Histopathological examination on biopsy confirmed recurrence of primary lacrimal sac melanoma.

**Discussion and conclusions:**

None has described the CT and MR findings of primary lacrimal sac melanoma so far. Typically, MR images show hyperintensity signal on T1-weighted images and hypointense signal on T2-weighted images owing to the paramagnetic properties of melanin. In contrast to previous reports and the present case, most cases do not present these typical signals. Thus, reporting such radiological findings is important to create awareness on variant images of primary lacrimal sac melanoma. This will reduce misdiagnosis and mistreatment.

## Background

Primary malignant melanoma of the lacrimal sac is an extremely rare condition. Yet, the lesion is highly malignant, can metastasize at a relatively early stage and patients with such lesions have poor prognosis. Early diagnosis is therefore essential. Notably, this condition is often misdiagnosed as chronic dacryocystitis. For accurate diagnosis, imaging examination is recommended to assess the severity of the disease, differentiate between tumors and inflammation and characterize tumor features. Here, we present a case of primary malignant melanoma of the lacrimal sac. We provide a summarize of the computed tomography (CT) and magnetic resonance imaging (MRI) findings and review previous literature on this condition.

## Case presentation

A 50-year-old female patient who had a mass in the lacrimal sac area for more than 3 months is presented in this report. The mass exhibited slow but progressive swelling. She experienced occasional epiphora and pain, without blood discharge or impaired vision. After 6 months, the patient underwent dacryocystectomy for the left lacrimal sac mass. Postoperative pathological examination confirmed the presence of malignant melanoma. Three months after the operation, a lump in the lacrimal sac area had gradually increased. Local recurrence was suspected and she was referred to ophthalmology department for further treatment.

**Fig. 1:**
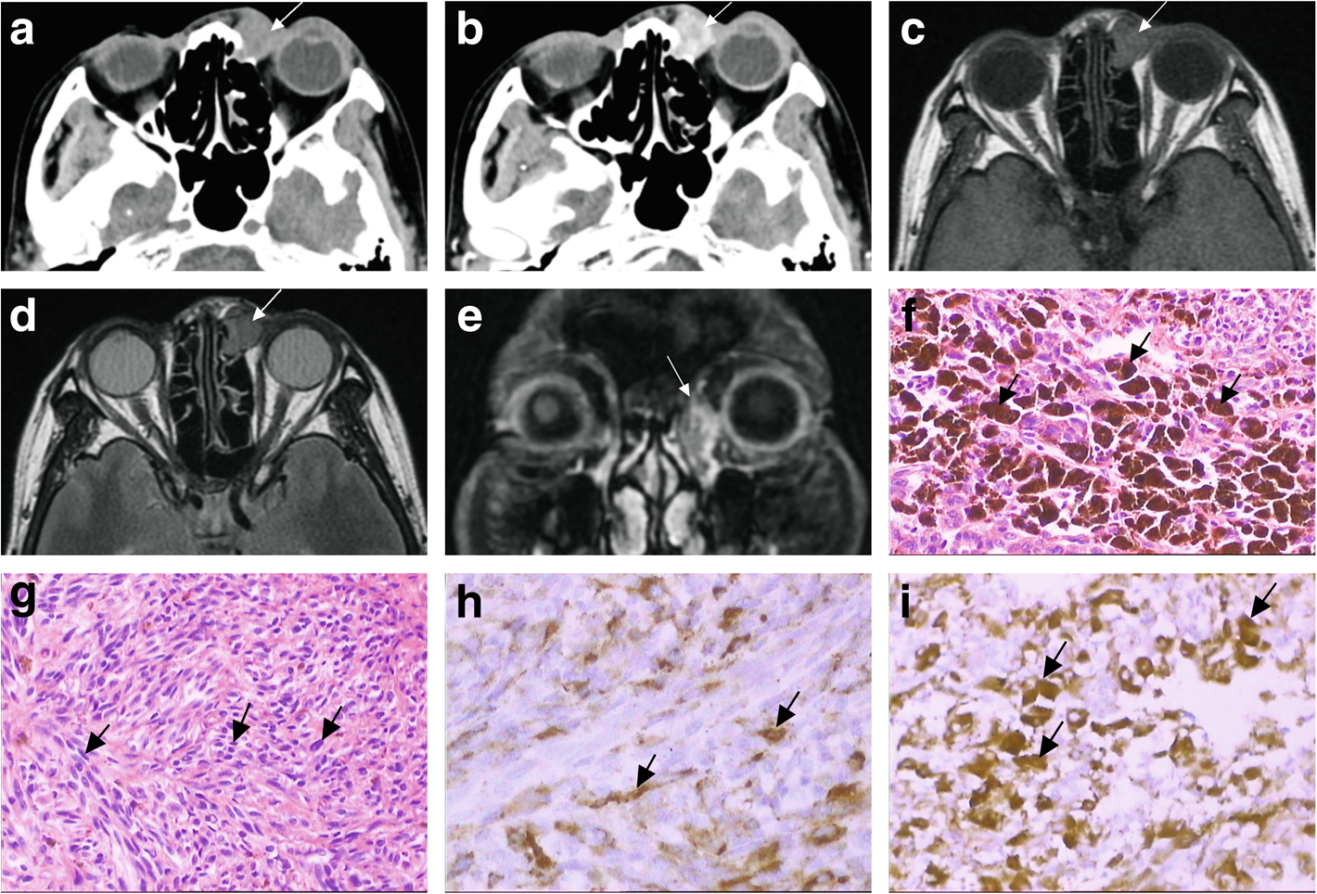
**a** Axial CT shows a partly well-defined soft mass in the fossa of left lacrimal sac. **b** An enhanced CT scan shows marked enhancement. **c** T1-weighted axial MRI shows an intermediate signal intensity mass. **d** T2-weighted axial MRI shows a iso-or hyper-signal mass. **e** Gadolinium-enhanced T1-weighted coronal MRI demonstrates a hyperintense, well-defined enhancing mass, extending into the nasolacrimal duct and anterior ethmoid sinus. **f** Photomicrography of the lacrimal sac melanoma showing heavy melanin pigmentation produced by tumor cells (hematoxylin-eosin, × 40). **g** These tumour cells producing higher magnification showing spindle and ovoid shaped tumor cells with inhomogeneous pigment granules (hematoxylin-eosin, × 20). **h** Immunohistochemical staining with HMB-45 showing positive staining of the tumor cells((immunohistochemical staining, × 40). **i** The tumour cells showing strong immunoreactivity to melanoma-specific antibody, Melan-A in the immunohistochemical examination. (immunohistochemical staining, × 20)

A physical examination revealed a scar from the previous operation. In addition, a 1.5 cm × 1.5 cm firm, localized and relatively clearly defined mass was observed in the left medial canthus. There was no cervical lymphadenopathy. A CT scan demonstrated a partially well-defined soft mass in the fossa of left lacrimal sac extending into the nasolacrimal duct and anterior ethmoid sinus (Fig. 1a). CT plain scan revealed that the lesion had a CT value of 49HU. The CT value of the tumor was similar to that of the extraocular muscle and wall of eyeball. Enhanced CT scan detected a CT value of 103HU for the arterial phase and 95HU for the venous phase (Fig. 1b). There was no eyeball involvement. Bone windows showed that the left nasolacrimal duct was enlarged and partial bone destruction of the nasolacrimal duct was confirmed. There was no regional or distant metastasis.

MRI plain scan demonstrated a partly well-demarcated mass of 1.2 cm × 1.3 cm × 2.3 cm in the lacrimal sac lesion. The tumors had infiltrated the septa orbital and subcutaneous adipose tissue. MRI revealed intermediate signal intensity on T1 and iso-or hyper-signal on T2 weighted images (Fig. 1c, d). The lesions displayed slight hyperintense signal on DWI. A dynamically enhanced MRI scan showed intense enhancement of the lesion margin and inhomogeneous enhancement of the lesion center (Fig. 1e).

A biopsy was obtained from the patient before the operation. Histopathological examination on the biopsy confirmed fusiform malignant tumoral cells with hyperchromatic nuclei. The tumor had high number of pigmented cells, inhomogeneous pigment granules and massive hemorrhage (Fig. 1f, g). Immunoreactivity to the S-100 protein, Melan-A, HMB-45, Nestin, and CD56 was positive (Fig. 1h, i). Therefore, a diagnosis of malignant melanoma was confirmed. Further examination of the whole body, including computed tomography scanning and B ultrasound revealed that there was no metastasis in the head and neck, chest, abdomen, and pelvis regions. Thus, total excision of tumor was performed. The patient refused to undergo radiotherapy and chemotherapy.

## Discussion and conclusions

Primary malignant melanoma of the lacrimal sac is a rare condition, with less than 50 cases reported over the past 90 years worldwide. The first case of this disease was published in 1926 in Russia by Muravleskin [1]. The incidence of primary lacrimal sac melanoma in lacrimal sac tumors ranges between 4 and 13% and it accounts for 0.7% of all ocular melanomas [2]. Melanoma cells are neuroectodermal tumors that originate from the neural crest. However, lacrimal sac epithelium does not contain melanocytic cells. It is speculated that malignant melanoma develops from melanin cells left in the epithelium of the lacrimal sac during the development of the embryonic system [3]. Based on a review of previous reports, the average age of patients at diagnosis is 59 years old (range of 27–81) with no significant sex predilection [1]. Primary melanomas of the lacrimal sac have a poor prognosis [2]. Clinically, its symptoms resemble those of dacryocystitis, leading to frequent misdiagnosis. Blood discharge (44%) and painless swelling (42%) are the most frequent symptoms of this condition. Due to its insidious onset, understanding the extent of invasion and early diagnosis are critical to facilitate timely intervention and improve the survival outcomes [1]. Although pathology and immunohistochemistry tests are needed to make a definitive diagnosis, CT and MRI imaging are also effective diagnostic tools for this condition [4]. Several reports on CT-scan for lacrimal sac melanoma show the presence of a soft lesion in the lacrimal sac fossa (Table 1). Similarly, our case presented isodensity and slightly high density on CT images, without calcification. Cystic change is uncommon.

**Table 1:** Description of CT findings in primary malignant melanoma of the lacrimal sac

case	references	CT density	extending area	bone destruction
1	Lloyd and Leone (1984) [5]	STM	^a^	^a^
2	Eide et al. (1993) [6]	STM	^a^	no
3	Owens et al. (1995) [7]	STM	ND and IM	^a^
4	Levine et al. (1996) [8]	STM	^a^	no
5	TY Malik et al. (1997) [9]	STM	^a^	no
6	Fishman and Ophir (1999) [10]	STM	ND, IM and NC	^a^
7	Lee et al. (2001)[11]	STM	ND and IM	^a^
8	Billing et al. (2003) [12]	STM	ND	no
9	Tello et al. (2004)[13]	STM	ND	no
10	Gleizal et al. (2005) [4]	STM	^a^	no
11	Nam et al. (2006)[14]	STM	ND and MOW	^a^
12	Lewis et al. (2006)[15]	STM	ND	no
13	Sitole et al. (2007)[16]	STM	^a^	posterior lacrimal duct wall
14	Heindl et al. (2008)[17]	STM	ND and NC	no
15	Li et al. (2012) [18]	STM	MOW	nasolacrimal canal and inferior turbinate
16	Maegawa et al. (2014) [19]	STM	ND and IM	^a^
17	Ren et al. (2014) [20]	STM	^a^	no
18	Pujari et al. (2014) [21]	isodense	^a^	anterior lacrimal crests infiltration
19	McGrath et al. (2016) [1]	STM	^a^	the lateral wall of upper nasal canal and anterior ethmoidal air cells
20	Kavoussi et al. (2016) [22]	STM	^a^	ethmoid sinuses
21	Present case	Isodense, slightly high	ND and AES	ND

*STM* soft tissue mass, *ND* nasolacrimal duct, *IM* inferior meatus, *NC* nasal cavity, *MOW* medial orbital wall, *AES* anterior ethmoid sinus, ^a^: not mentioned

In the early stage, imaging examination showed round, well-defined and homogeneous density lesions without orbital or nasal sinus involvement. Nasolacrimal duct is easily involved [4], according to previous literatures on CT images, the lesions extend into the nasolacrimal duct without nasolacrimal duct bone destruction, accounting for 50% (10/20) of CT images (Table 1), after which they invade the surrounding bone and soft tissue. In the late stage, the lesions tended to be irregular and ill-defined masses with invasion of nasolacrimal duct, orbital and sinus [22]**.** CT is more sensitive to bone destruction than MRI [4]**.** Non-epithelial lesions appear to cause lower bone destruction than epithelial tumors. In accordance with previous imaging findings, only five cases mentioned bone destruction on CT imagines (5/20) (Table 1). Because our case is a relapsing primary malignant melanoma of the lacrimal sac after surgery, bone destruction may result from the previous dacryorhiocydtostomy. The operation was done in other hospitals. There was no CT before the operation. Unfortunately, we don’t know the exact cause of bone destruction.

Of note, CT is not sufficient for tumor diagnosis because CT values do not show the number of melanin granules. In contrast, MRI images provide effective diagnosis for malignant melanoma. MRI has a high soft-tissue resolution, making it the best imaging technique for evaluating malignant melanoma currently. Typically, MR images show the presence of hyperintensity signal on T1-weighted images and hypointense signal on T2-weighted images owing to the paramagnetic properties of melanin in most other parts of malignant melanomas. But this condition is rare in malignant melanoma of lacrimal sac. To the best of our knowledge, only six case reports have been published describing the MRI radiological features of this condition. Four cases (4/7) (including our case) show intermediate signal intensity on T1 weighted image, and only one case presented with typical signals on T2 weighted images (Table 2).

**Table 2:** Description of MR findings in primary malignant melanoma of the lacrimal sac

case	references	MR features
1	Billing et al. (2003) [12]	intermediate signal intensity on T1 and T2 weighted images, enhanced with intravenous gadolinium
2	Tello et al. (2004) [13]	intermediate signal intensity on T1 weighted images and hyperintensity on T2 weighted images
3	Lewis et al. (2006) [15]	iso-or hyper-signal on T1 weighted images
4	Li et al. (2012) [18]	lowerintensity on T1 weighted images and highintensity on T2 weighted images, gadolinium-enhanced T1-weighted highintensity
5	Maegawa et al. (2014) [19]	hypointense on T2
6	Kavoussi et al. (2016) [22]	intermediate signal intensity on T1 and T2 weighted images
7	Present case	intermediate signal intensity on T1 weighted images and iso-or hyper-signal on T2 weighted images

The characteristic MR signals are based on histologic features. The higher the melanin content, the more typical the signal is. In our case, massive hemorrhage and inhomogeneous pigmentation may precipitate the untypical signals, and since after only 3 months theretumor blood supply and structure could still be some post-surgical changes in MR signals. This untypical signal may lead to misdiagnosis of amelanotic melanoma, which accounts for 2% of all cases of melanoma [23]. The malignant melanoma of the lacrimal sac contains fewer melanocytes than uveal melanoma. Compared with other parts of orbital malignant melanoma images, fewer cases present with the typical paramagnetic signals. Less pigmentation and intratumoral hemorrhage may cause untypical MR signals in the lacrimal sac, and therefore they should be considered during diagnosis. Malignant melanoma of the lacrimal sac may present intermediate signal intensity on T1 and iso-or hyper-signal on T2 weighted images in most cases. Intravenous gadolinium provides more information on tumor blood supply and structure [22]. However, only one case mentioned moderated image enhancement of lesions excluding our case. The patient in this report exhibited marked enhancement of lesions, suggesting hypervascular neoplasms. According to previous studies, many orbital melanomas contain moderate and intense enhancement lesions following the administration of contrast material [24]. Contrast enhancement may improve the detection. Unfortunately, contrast enhanced cases are few and thus the characteristics of melanoma of lacrimal sac cannot be summarized.

The differential diagnosis of melanoma in the lacrimal sac considers primary epithelial neoplasms (85.6%), malignant lymphoma (7.8%), neural tumors, inflammatory lesions and metastatic tumor [25]. Cases with well-defined lesions extending into the nasolacrimal duct with minimal bone destruction are likely to be nonepithelial neoplasms. Most lacrimal sac tumors present intermediate signal intensity on T1 and T2 weighted images. Typical signals of malignant melanoma of lacrimal sac are rare, so high scrutiny is required to identify them. Imaging can diagnose cystic mass [26]. Imaging examination can effectively identify the location, size, extent of lesions, and invasion of adjacent soft tissue. The CT and MRI examinations are suitable for determining tumor recurrence during patient follow-up. Imaging examination provides more reliable information to guide decision-making in the choice of clinical treatment. Definite diagnosis should be made by pathologic evaluation and immunohistochemistry. A limitation of this case report is that imaging was only performed at the relapsing stage, and thus bone destruction may result from tumor development or from the initial surgery (or both).

To date, none has summarized the CT and MR findings on primary lacrimal sac melanoma. Based on previous reports and our case, most cases do not present typical signals. Thus, reporting such radiological findings is important to create awareness on variant images of primary lacrimal sac melanoma. This will reduce misdiagnosis and mistreatment.

## Data Availability

Not applicable.

## Abbreviations

AES: Anterior ethmoid sinus
CT: Computed tomography
IM: Inferior meatus
MOW: Medial orbital wall
MRI: Magnetic resonance imaging
NC: Nasal cavity
ND: Nasolacrimal duct
STM: Soft tissue mass
